# SNUH methylation classifier for CNS tumors

**DOI:** 10.1186/s13148-025-01824-0

**Published:** 2025-03-12

**Authors:** Kwanghoon Lee, Jaemin Jeon, Jin Woo Park, Suwan Yu, Jae-Kyung Won, Kwangsoo Kim, Chul-Kee Park, Sung-Hye Park

**Affiliations:** 1 Department of Pathology, Seoul National University College of Medicine, 101 Daehak-ro, Jongno-gu, Seoul, Republic of Korea; 2 Interdisciplinary Program in Bioinformatics, Seoul National University, Seoul, Republic of Korea; 3 Department of Pathology, Yonsei University College of Medicine, Seoul, Republic of Korea; 4 Department of Transdisciplinary Medicine, Seoul National University Hospital, Seoul, Republic of Korea; 5 Department of Medicine, Seoul National University, Seoul, Republic of Korea; 6 Department of Neurosurgery, Seoul National University College of Medicine, Seoul, Republic of Korea; 7 Neuroscience Research Institute, Seoul National University College of Medicine, Seoul, Republic of Korea

**Keywords:** Methylation, Brain tumors, Classification, Next-generation sequencing, Targeted therapy

## Abstract

**Background:**

Methylation profiling of central nervous system (CNS) tumors, pioneered by the German Cancer Research Center, has significantly improved diagnostic accuracy. This study aimed to further enhance the performance of methylation classifiers by leveraging publicly available data and innovative machine-learning techniques.

**Results:**

Seoul National University Hospital Methylation Classifier (SNUH-MC) addressed data imbalance using the Synthetic Minority Over-sampling Technique (SMOTE) algorithm and incorporated OpenMax within a Multi-Layer Perceptron to prevent labeling errors in low-confidence diagnoses. Compared to two published CNS tumor methylation classification models (DKFZ-MC: Deutsches Krebsforschungszentrum Methylation Classifier v11b4: RandomForest, 767-MC: Multi-Layer Perceptron), our SNUH-MC showed improved performance in F1-score. For ‘Filtered Test Data Set 1,’ the SNUH-MC achieved higher F1-micro (0.932) and F1-macro (0.919) scores compared to DKFZ-MC v11b4 (F1-micro: 0.907, F1-macro: 0.627). We evaluated the performance of three classifiers; SNUH-MC, DKFZ-MC v11b4, and DKFZ-MC v12.5, using specific criteria. We set established ‘Decisions’ categories based on histopathology, clinical information, and next-generation sequencing to assess the classification results. When applied to 193 unknown SNUH methylation data samples, SNUH-MC notably improved diagnosis compared to DKFZ-MC v11b4. Specifically, 17 cases were reclassified as ‘Match’ and 34 cases as ‘Likely Match’ when transitioning from DKFZ-MC v11b4 to SNUH-MC. Additionally, SNUH-MC demonstrated similar results to DKFZ-MC v12.5 for 23 cases that were unclassified by v11b4.

**Conclusions:**

This study presents SNUH-MC, an innovative methylation-based classification tool that significantly advances the field of neuropathology and bioinformatics. Our classifier incorporates cutting-edge techniques such as the SMOTE and OpenMax resulting in improved diagnostic accuracy and robustness, particularly when dealing with unknown or noisy data.

**Supplementary Information:**

The online version contains supplementary material available at 10.1186/s13148-025-01824-0.

## Background

Accurate tumor classification, including subtyping and grading, is crucial for diagnosis and guiding clinical treatment strategies. Neuropathologists have traditionally employed diverse tools, such as histopathology, immunohistochemistry, and molecular genetic tests like fluorescence in situ hybridization (FISH), and Sanger sequencing, to classify central nervous system (CNS) tumors with genetics integrated diagnosis. While these methods have been invaluable, they sometimes face challenges due to ambiguous or heterogeneous morphologies, as well as shared immunohistochemical markers and molecular genetic abnormalities across CNS tumors [1]. The introduction of next-generation sequencing (NGS) marked a significant advancement in diagnostic precision, particularly for specific brain tumors, by integrating comprehensive molecular genetics into pathology assessments [2–4]. As NGS was being established in diagnostic neuropathology, methylation profiling emerged as a complementary approach, offering additional insights into CNS tumor classification [5].

The updated 2021 WHO Classification of Tumors of the CNS (WHO 2021) identified molecular genetic and epigenetic study, particularly methylation profiling as a crucial tool for accurate diagnosis and classification [6]. These molecular approaches have proven particularly valuable in enhancing the complex subclassification of certain brain tumors, such as ependymomas and medulloblastomas.

Ependymomas, which arise from ependymal cells lining the cerebral ventricles and the spinal cord’s central canal. Neuropathologists have established that subclassification according to their anatomical locations: supratentorial (ST), posterior fossa (PF), and spinal (SP). This classification system was initially proposed based on distinct methylation profiles observed in each subtype [7, 8]. Upon further analysis, the difference was not limited to anatomical location but also includes distinct genetic profiles. This insight enabled the stratification of ependymoma based on both genotypic characteristics and histological grade. Notably, this refined classification system for ependymomas demonstrates a strong correlation with the biological behavior observed in patients, thus providing a more comprehensive and clinically relevant approach to tumor categorization. ST-ependymomas often exhibit specific genetic alterations, particularly *ZFTA* fusion and *YAP1* fusion, while, PF-ependymomas typically lack gene-level changes [8]. Instead, PFA-ependymoma is marked by EZHIP overexpression and at the same time H3K27me3 loss. For SP-ependymomas, methylation studies have proven particularly beneficial. These tumors often harbor chromosome 22 copy loss, *NF2* gene copy loss, or *NF2* mutations, which can be challenging to detect using conventional molecular tests such as Sanger sequencing. The methylation classifier outperforms conventional FISH or NGS methods by identifying MYCN-amplified spinal ependymoma even when no gene-level amplification is detected. By integrating methylation profiling with established diagnostic methods, neuropathologists can now achieve a more comprehensive and accurate classification of CNS tumors, especially in cases where traditional approaches may be inconclusive or limited.

Medulloblastoma, the most common malignant pediatric brain tumor, comprises four molecular subgroups: WNT, SHH, Group 3, and Group 4. These subgroups differ in genetic profiles and clinical outcomes, making molecular classification crucial for treatment planning. However, NGS methods using targeted gene panels often struggle to distinguish Group 3 and Group 4 medulloblastomas, which frequently lack specific gene-level alterations. Instead, these subtypes are characterized by epigenetic changes, including structural variations and enhancer hijacking. Consequently, comprehensive molecular profiling approaches, such as DNA methylation analysis, are essential for the accurate classification of medulloblastoma subgroups [3, 9–11].

Advances in machine learning have revolutionized brain tumor methylation classification, with the Deutsches Krebsforschungszentrum Methylation Classifier (DKFZ-MC) emerging as a notable innovation. This classifier utilizes RandomForest methods to select 10,000 probes for feature selection, creating a robust model for tumor classification [12]. The DKFZ-MC requires a methylation class score above 0.9 for confident tumor classification. A calibration model enhances the classifier’s accuracy. Samples scoring below 0.9 undergo further histopathological analysis for tumor classification, ensuring a comprehensive diagnosis. This innovative method combines RandomForest techniques for probe selection with a Multi-Layer Perceptron for extracting performance metrics [13]. While the classifier’s code is not publicly accessible, limiting accessibility, the DKFZ-MC has played a crucial role in advancing our understanding of brain tumor classification.

As research continues, such advanced classification tools are expected to further enhance personalized treatment strategies and patient outcomes in brain tumor management.

Traditional brain tumor classification methods often operate under a closed-set recognition framework, assuming all possible tumor classes are known and represented in the training data. While effective for classifying known tumor types, this approach falls short in real-world scenarios where novel, previously unseen tumor types may emerge.

This study introduces open-set recognition to CNS tumor classification, addressing the evolving landscape of neuro-oncology. The innovative approach allows our classifiers to not only accurately identify and categorize known tumor types but also recognize samples that do not match any known class, assigning unmatched samples to an “unknown” category. This open-set recognition is particularly crucial in neuro-oncology, where new tumor subtypes are continually being discovered. By implementing this method, we significantly reduce the risk of misclassifying novel or atypical tumors as known types, a critical improvement that can prevent potential misdiagnoses and inappropriate treatment decisions. The ability to identify “unknown” cases not only enhances diagnostic accuracy but also flags samples that may represent new tumor entities, potentially contributing to the advancement of CNS tumor classification. This approach aligns closely with clinical realities, where atypical cases are not uncommon, and provides a more robust framework for handling the complexities of brain tumor diagnostics in real-world settings.

This study introduces ‘Augmented Open Set Detection Methylation Classifier’ of our Seoul National University Hospital (referred to as SNUH-MC). Our research aims to elucidate the decision-making process of SNUH-MC, validate its efficacy using diverse methylation array datasets, including our institutional data, demonstrate the impact of methylation profiling on CNS tumor pathological diagnosis, conduct a comparative analysis between DKFZ-MC versions v11b4 and v12.5.

## Materials and methods

### Data collection

We compiled 11 diverse datasets for our study sourced from multiple open databases. These datasets, containing raw methylation data specific to brain tumors, are detailed in Supplementary Table 1 [12, 13]. ‘Train Set’ was used for model training and establishing baseline performance. We used three test sets. ‘Test Set 1’ contained identical methylation classes as the Train Set, ensuring consistency in initial comparisons. ‘Test Set 2’ introduced new and diverse brain tumor methylation data with varying noise levels, assessing model robustness and predictive capabilities in challenging scenarios. ‘Test Set 3 (Sarcoma Dataset)’ comprised non-brain tumor patient data, specifically designed to evaluate the model’s open-set recognition capabilities.

The Train Set, Test Set 1, and Test Set 2 share 91 methylation classes. The distribution of patient samples across these classes is visualized in Supplementary Fig. 1, providing a clear representation of sample distribution and supporting our comparative analysis. This structured approach allows us to establish baseline performance (Train Set and Test Set 1), assess robustness in diverse scenarios (‘Test Set 2’),’ and evaluate open-set recognition capabilities (Test Set 3). By utilizing these varied datasets, we ensure a thorough and multifaceted evaluation of our model’s performance across different conditions and data types.

### Sample preparation and EPIC array data generation

In the selection of 193 cases of CNS tumors for methylation array analysis, two neuropathologists specifically targeted cases with diagnostic challenges based on histopathology and NGS results. Within the SNUH dataset, neuropathologists ensured microdissection targeting tumor cells from formalin-fixed and paraffin-embedded (FFPE) blocks or fresh frozen (FF) tissues.

Subsequent DNA extraction was performed using Maxwell^®^ RSC kits (AS1880 for FF and AS1450 for FFPE) following the manufacturer’s specified instructions. All methylation data at SNUH were obtained through experiments using the Infinium MethylationEPIC v1.0 kit by Illumina.

### Data preprocessing, batch effect correction, and model development

**Fig. 1:**
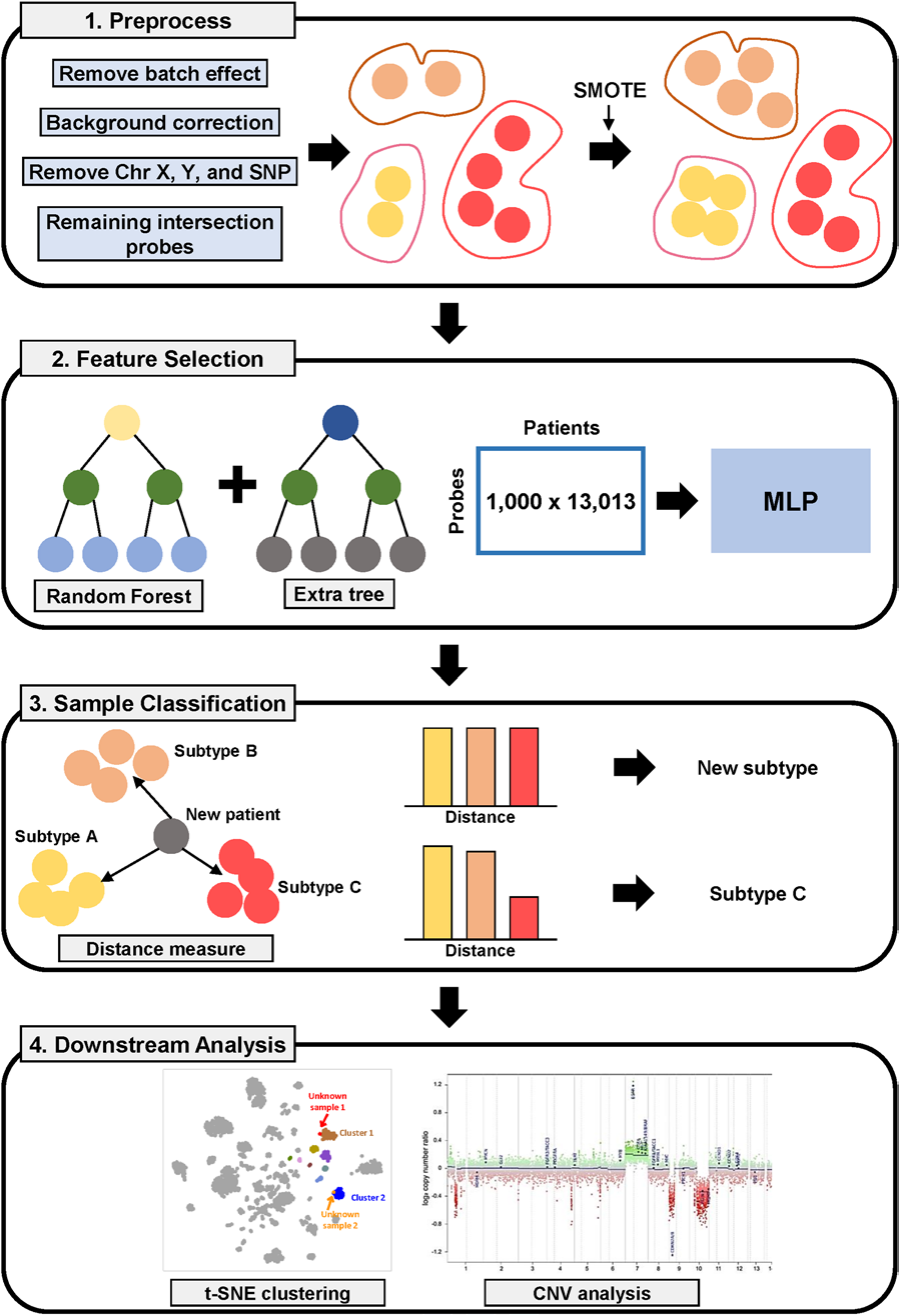
Workflows of SNUH-MC. The SNUH-MC’s workflow encompassed three primary stages: ‘Preprocessing,’ ‘Feature Selection,’ and ‘Sample Classification.’ Initially, preprocessing was executed using the ‘DKFZ-MC’ methodologies. Post probe selection, the SMOTE algorithm was employed, resulting in an oversampling from the original 2801 samples to 13,013 samples. Feature selection was conducted using the RandomForest classifier to identify the top-ranked probes. For the construction of the classification model, the MLP method was applied, based on the top 2000 probes. Additionally, to enhance open-set recognition capabilities, SoftMax values were integrated with the OpenMax algorithm [14]. In the downstream analysis, the visualization step could be undergone. tSNE plot was used to locate where the unidentified sample was clustered. CNV plot can be generated using the red and green signal intensity of probes (SNUH-MC: Seoul National University Hospital Methylation Classifier; DKFZ-MC: Deutsches Krebsforschungszentrum Methylation Classifier; SMOTE: Synthetic Minority Over-sampling Technique; MLP: Multi-Layer Perceptron; CNV: Copy Number Variation)

The raw methylation data underwent rigorous processing, including quality control, normalization, and batch effect correction (Fig. 1). Our batch effect correction methodology employed the linear model-based approach provided by the removeBatchEffect function from the limma package in R. This process involved three key steps: (1) Data Transformation: We applied a log2(methy + 1) transformation to stabilize variance across the dataset, which is crucial for high-throughput methylation array data. (2) Batch Effect Removal: The removeBatchEffect function was applied to the log-transformed data, fitting a linear model to each CpG site with the batch variable as a covariate. The estimated batch effect was then subtracted from the data. 3) Inverse Transformation: We applied 2^ to the output, reversing the log transformation and restoring the data to its original scale, now with batch effects removed.

To quantify the effectiveness of our batch correction, we conducted principal component analysis (PCA) before and after the correction. This analysis revealed a significant reduction in batch-related variation, with 15% of batch-associated variance eliminated. Supplementary Fig. 2 illustrates the PCA results, demonstrating the reduction in batch-related clustering. Following batch correction, feature selection was performed using a combination of statistical methods and domain knowledge, identifying the most informative methylation probes for CNS tumor classification. This comprehensive preprocessing approach ensured that our subsequent analyses were based on high-quality data with minimized technical batch effects, allowing for more reliable biological interpretations and robust tumor classification.

SNUH-MC classifier was developed using a deep learning architecture, specifically a Multi-Layer Perceptron (MLP) with integrated OpenMax for open-set recognition. The OpenMax component aimed to prevent labeling errors in low-confidence diagnoses by introducing an “unknown” class during training.

In our dataset, we observed an unequal distribution of labeled training data across different tumor types, a common challenge in medical datasets known as data imbalance. This imbalance is clearly illustrated in Supplementary Fig. 1, which shows the varying sample sizes for each tumor class. To address the data imbalance in CNS tumor datasets, we implemented Synthetic Minority Over-sampling Technique (SMOTE), generating synthetic samples for underrepresented tumor classes. This approach created a more balanced training dataset, mitigating potential biases toward common tumor types.

Our model training combined and semi-supervised learning techniques, leveraging both labeled and unlabeled data. We conducted extensive hyperparameter tuning and cross-validation to optimize performance and generalizability. This comprehensive strategy aimed to develop a robust methylation classifier capable of accurate identification across diverse CNS tumor types, regardless of their prevalence in the original dataset.

We initially processed the methylation data using methods similar to those employed by the ‘DKFZ-MC14.’ To further refine our dataset and enhance the reliability of our analysis, we implemented additional probe filtering steps. Specifically, we removed probes falling into the following four categories:

1. Probes situated on the sex chromosomes: Methylation patterns on X and Y chromosomes differ significantly between males and females, which could introduce confounding effects in our analysis of autosomal methylation differences.
2. Probes lacking unique alignment to the hg19 reference genome: Non-uniquely aligned probes were removed to ensure specificity. Probes aligning to multiple genomic locations can lead to ambiguous methylation signals, potentially confounding the interpretation of site-specific methylation status.
3. Probes containing single nucleotide polymorphisms (SNPs): SNP-containing probes were excluded because genetic variations can affect probe binding efficiency. This can result in biased methylation measurements that do not accurately reflect the true methylation status of the target site, compromising data reliability.
4. Probes not incorporated in the EPIC chip: We removed probes absent from the EPIC chip to ensure compatibility and comparability across different methylation array platforms. This step facilitates the application of our classifier to data generated from both older and newer methylation array technologies.

By implementing these filtering criteria, we aimed to create a robust and reliable set of methylation probes, minimizing potential sources of technical bias and focusing on high-quality, interpretable methylation signals for our CNS tumor classification model.

Subsequently, 428,230 probes were utilized across the ‘Train set,’ ‘Test sets 1 and 2,’ and ‘SNUH data’ for feature selection and constructing the classification model. This approach ensured uniformity and practicality across various datasets, significantly contributing to the robustness of the model’s performance evaluation.

The initial feature selection step involved using the ‘Synthetic Minority Over-sampling Technique (SMOTE)’ algorithm [15]. This technique increased the original 2801 samples to 13,013 samples, equalizing the number of samples across classes (Supplementary Fig. 3). After oversampling, a RandomForest classifier was employed for feature selection with specific parameters: maximum features set to the square root of the total number of features (max_features = ‘sqrt’), and the number of trees in the forest set to 4000 (n_estimators = 4000). This approach enabled obtaining feature importance values and selecting the top-ranked probes essential for the method’s functionality.

In constructing the classification model, a ‘Multi-Layer Perceptron’ approach was adopted [13], focusing on the top 1000 probes selected based on their feature importance values. The model utilized a leaky Rectified Linear Unit activation function and Stochastic Gradient Descent as an optimization tool. The network architecture implemented a significant hidden layer comprising 80,000 units, and early stopping techniques were incorporated to mitigate overfitting.

SoftMax values were utilized in conjunction with the OpenMax algorithm to facilitate the open-set recognition. When there are $K$ known classes with a given input data $x$, the network produces logits ${z}_{k}$ for each $k$. The SoftMax probability for class k is given by following equation.

$$P\left(k|x\right)= \frac{\text{exp}({z}_{k})}{\sum_{j=1}^{K}\text{exp}({z}_{j})}$$

This integration allowed the model to make predictions regarding new labels while maintaining classification accuracy for known classes. The OpenMAX algorithm works by recalibrating the output layer of a neural network, expanding the SoftMax output with a label indicating “no known category.” This is achieved by estimating the probability that the input does not belong to a known class. Let ${f}_{k}\left(x\right)$ represent the recalibrated score for each class k after applying OpenMAX. The probability of the input belonging to each class k $P^{\prime } \left( {k{|}x} \right)$ and the “unknown” class u $P^{\prime } \left( {u{|}x} \right)$ is then given by following equation.

$${P}^{\prime}\left(k|x\right)=\frac{{f}_{k}\left(x\right)}{\sum_{j=1}^{K}{f}_{j}\left(x\right)+{f}_{u}\left(x\right)}$$

$${P}^{\prime}\left(u|x\right)=\frac{{f}_{u}\left(x\right)}{\sum_{j=1}^{K}{f}_{j}\left(x\right)+{f}_{u}\left(x\right)}$$

OpenMax computes the distance vector between the input features $x$ and the average activation vector for each known class. These distances help us understand how far the input is from known class features. Specifically, for each class $k$, the distance between input features and the mean activation vector ${\mu }_{k}$ is considered. OpenMax then models the tails of these distance distributions for each class using a Weibull distribution to estimate the likelihood that an input might belong to an unknown class.

The recalibrated score for each class $k$ is computed with the following equation:

$${f}_{k}\left(x\right)=p\left(k|x\right)*\left(1-{w}_{k}\left(x\right)\right)$$

The Weibull-derived probability is calculated as follows:

$${w}_{k}\left(x\right)=\text{exp}\left(-\left(\frac{dis(x,{\mu }_{k})}{{\sigma }_{k}}\right)\right)$$

Here, distinct function is the calculating distance between input features and mean activation vector for each class and ${\sigma }_{k}$ is the scale parameter of the Weibull distribution for each class.

The “unknown” score ${f}_{u}\left(x\right)$ is then computed as the following equation:

$${f}_{u}(x) = \sum_{k=1}^{K}P(k|x)*{w}_{k}\left(x\right)$$

Recalibration includes SoftMax probability correction. The OpenMax layer considers the possibility that the input comes from an unknown class and recalculates these probabilities by reducing the SoftMax score of a known class proportional to its distance from the class mean and the modeled Weibull distribution, and then assigning the reduced probability mass to a new “unknown” class label. The final predicted class is determined by selecting the class with the highest recalibrated probability, including the possibility of the “unknown” class:

$\widehat{y}= \underset{k\in \{1,...,K,u}{\text{argmax}}P^{\prime}(k|x)$ This novel approach ensured robustness in classifying known categories while extending the model’s capability to recognize and classify previously unseen classes, greatly contributed to the adaptability and versatility of SNUH-MC.

### Histopathological diagnosis

Many brain tumors lack consistent morphological patterns and may exhibit varied or ambiguous morphologies within the same tumor type, depending on their grade or differentiation. For instance, ‘glioblastoma, IDH-wildtype (GBM, IDH-wt),’ ‘diffuse midline glioma, H3 K27-altered,’ ‘diffuse hemispheric glioma, H3 G34-mutant,’ and ‘diffuse pediatric-type high-grade glioma, H3-wt and IDH-wt’ can display an overlapping or broad spectrum of morphological features, ranging from uniform mildly pleomorphic small cells to primitive small cells with a high nucleocytoplasmic ratio, or even extensively large multinucleated giant cells. In cases of ‘diffuse astrocytomas, IDH-mutant,’ there might be a mixture or collision of cells resembling oligodendroglioma and astrocytoma [16]. Consequently, relying solely on histopathology for diagnosis might result in misinterpretation, necessitating the use of immunohistochemical or genetic markers specific to each CNS tumor for accurate diagnosis.

CNS tumors, especially those without distinct genetic markers, often require methylation classifiers for accurate diagnosis. Relying solely on a specific genetic alteration across different tumor types is less reliable and cannot specifically differentiate between clinically relevant tumor classes, as such alterations lack the specificity needed for diagnosing distinct tumor types. For example, *BRAF* V600E-mutant gliomas encompass a spectrum of tumor types, including ‘diffuse low-grade gliomas, MAPK pathway-altered,’ pleomorphic xanthoastrocytoma, pilocytic astrocytoma, ganglioglioma, and desmoplastic infantile astrocytoma/ganglioglioma, or epithelioid GBM [17–19].

Moreover, methylation classifiers play a significant role in achieving accurate diagnosis, subtyping of tumors, and assessing copy number aberrations and MGMT promoter methylation simultaneously.

### DNA extraction and next-generation sequencing (NGS) study

In the process of isolating DNA from tumor samples, representative tumor areas with a minimum of 90% purity were identified within FFPE sections. These areas were delineated for macrodissection.

DNA extraction was performed using the Maxwell^®^ RSC DNA FFPE Kit (AS1450; Promega, USA), according to the manufacturer’s instructions. NGS was performed with a custom panel developed at Seoul National University Hospital (SNUH) for CNS tumor diagnosis, named the FiRST brain tumor panel. For sequencing, the Hi-Output NextSeq550Dx sequencing platform was used. Panel version 2 encompassed 172 genes, while Panel version 3 comprised 207 genes and Panel 3.1 had 228 genes in addition to including 20, 52, and 155 fusion genes, respectively [20].

The sequencing data underwent thorough analysis via the SNUH FiRST Brain Tumor Panel Analysis pipeline. Initially, quality control was conducted on the FASTQ file, ensuring that only data meeting specific criteria were included in subsequent analyses. The criteria are general and typical quality controls for FASTQ files. These include:

- Base quality scores: Ensuring that the sequencing reads meet a minimum quality threshold.
- Read length: Checking that reads are of expected length.
- GC content: Verifying that the GC content is within an acceptable range.
- Sequence duplication levels: Assessing the level of PCR duplicates.
- Overrepresented sequences: Identifying any abnormally frequent sequences that might indicate contamination or bias.

Paired-end alignment to the hg19 reference genome was executed using BWA-mem and adhering to GATK Best Practice guidelines [21]. This alignment step resulted in an ‘analysis-ready BAM’ file, followed by a second quality control check to assess the suitability for further variant calling. Variations such as single nucleotide variations (SNV), insertions and deletions (InDel), copy number variations (CNV), and translocations underwent analysis using at least two different tools, comprising both in-house and open-source software. Notably, open-source tools like GATK UnifiedGenotyper, SNVer, and LoFreq were employed for SNV/InDel detection, while tools such as Delly and Manta were utilized for translocation discovery. Purity estimation was conducted using THetA2, and CNV calling relied on CNVKit [8].

### Categorization and visualization of methylation profiling results

To obtain methylation class results from each classifier, we applied our unknown samples to the SNUH-MC and submitted them to ‘https://www.molecularneuropathology.org/mnp/’. The results were then grouped based on calibrated score ranges (score ≥ 0.9, 0.9 > score ≥ 0.5, score < 0.5) guided by insights from existing literature [22].

The 0.5 cutoff is used for distinguishing subclasses within methylation class families, particularly in cases like IDH-wt gliomas where subclass differentiation is crucial. The 0.9 cutoff, the higher threshold was chosen to indicate a very high confidence in classification.

These boundaries align with widely recognized standards, ensuring our results are comparable and comprehensible to both clinical and research audiences. While some cutoffs were initially chosen arbitrarily, their effectiveness has been validated in clinical settings, such as in IDH-mutant and 1p/19q-codeleted oligodendroglioma cases.

The term “methylation classifier” in our study refers to the initial classification step that uses DNA methylation data. However, the final classification is the result of an integrated decision-making process that we call “Decisions.” This process combines: the methylation-based classification, histopathological findings, immunohistochemical (IHC) findings, NGS data, and clinical information.

In cases where the methylation classifier yields a calibrated score below 0.9, we initially assign a “Likely match” status. However, this is not the final classification. We then consider the integrated information from the other sources mentioned above to determine the reliability and accuracy of the classification. If the additional data sources (histopathology, IHC, clinical information, and NGS) support the methylation-based classification, we maintain the classification even with a lower calibrated score. This integrated approach allows us to leverage multiple lines of evidence for more robust and accurate tumor classification. Therefore, our final classifier is indeed combined or integrated, rather than a purely methylation-based one. These assessments were denoted as ‘Decisions’ and are detailed in Table 1.

**Table 1:** Detailed descriptions of ‘decisions’

Decisions	Description
Match	When the calibrated score reaches 0.9 or above, it aligns with the results derived from histopathology, clinical information, CNS tumor-targeted NGS panel results, and the initial pathological diagnosis
Likely match	When the calibrated score falls below 0.9, histopathology, clinical information, CNS tumor-targeted NGS panel results, and the initial pathological diagnosis can be integrated to decide whether the methylation class is reliable. But keep in mind that the diagnosis can be changed
Uncertain	The methylation classifier’s result remains uncertain, irrespective of the calibrated score
No match	The methylation classifier’s result is incorrect
Control (No match)	It is categorized as normal control, which can occur when DNA is extracted from regions with very low tumor purity or normal brains

For visualization purposes, bar plots were generated using the R package ‘ggplot2 (v3.4.3)’, while alluvial plots were created using the ‘ggalluvial (v0.12.5)’. All plot generation processes were executed within the R system, version 4.2.0. This visual representation allowed for a clear understanding and comparison of the methylation classifier’s results with other diagnostic criteria, helping assess the reliability and consistency of the classification results.

## Results

To evaluate SNUH-MC performance, we conducted a comprehensive comparative analysis across various datasets (Supplementary Table 1). Our evaluation strategy was thorough and structured, aiming to assess SNUH-MC’s performance on diverse test datasets and its ability to differentiate among brain tumor data. Aligning our evaluation methodology with the established results presented by Capper et al. [12] ensured a standardized and comparable analysis.

Replicating the original code for ‘Test Data Set 2’ enabled direct comparison and contributed to the reliability and validity of the evaluation process. Additionally, when evaluating a methylation classifier’s ability to identify noisy data, sorting samples based on their calibrated scores represented a meticulous approach for detailed performance comparisons.

This rigorous evaluation, which included various classification scenarios and comparisons with existing benchmarks, offered a holistic understanding of SNUH-MC’s robustness and efficiency in distinguishing distinct datasets and recognizing noise, adding significant credibility to our evaluation.

### Performance evaluation

We compared the performance of our SNUH Methylation Classifier (SNUH-MC) with that of the DKFZ Methylation Classifier (DKFZ-MC) using Test Data Set 1. This comparison was conducted statistically by analyzing the calibrated scores from both classifiers on the same dataset. Our intention was to assess how SNUH-MC performs relative to DKFZ-MC, providing insights into the strengths and potential areas for improvement of our classifier.

We assessed the alignment between histopathological and methylation-based classifications of brain tumors. Using 2021 updated WHO classification definitions as the histopathological standard, we compared the results of methylation-based classification to the histopathological classification for each tumor sample.

Both classifiers categorized the sample into 92 classes, comprising the original 91 classes plus an additional ‘unknown’ label. We employed the F1 score as our primary metric, given its balanced representation of precision and recall, particularly valuable for imbalanced datasets.

For DKFZ-MC classification were made only when predictions exceeded a 0.9 confidence threshold. Below this threshold, clinical experts determined the class using histological images and methylation data, sometimes concluding ‘not applicable.’ SNUH-MC, with its internal mechanism for identifying ‘unknown’ classes, assigned and ‘unknown’ label under similar circumstances. We treated the SNUH-MC’s ‘unknown’ label and DKFZ-MC’s ‘not applicable’ designation as equivalent resulting in both models evaluating 92 classes, including this additional category. This approach allowed for a fair and comprehensive comparison of the two classifiers, accounting for their handling of uncertain cases and providing insights into their performance across individual classes and overall accuracy.

Given the importance of reducing misclassification in medical diagnoses, the models’ ability to confidently predict specific diseases and minimize ‘unknown’ classifications for patients with a particular condition is critical. To assess this aspect, we created a ‘Filtered Test Data Set 1,’ which excluded samples labeled as ‘unknown’ or ‘not applicable,’ thereby focusing on the 91 known classes for performance comparison. We also compared performance across the full Test Data Set 1, using all 92 labels. For ‘Filtered Test Data Set 2’, which lacked pathologically determined results, we modified model to include an unknown class by assigning the lowest probability to the unknown class.

To provide a comprehensive assessment, we calculated both macro and micro F1 scores. The macro F1 score, an average of F1 scores computed independently for each class, ensures equal treatment of all classes regardless of size. Conversely, the micro F1 score aggregates contributions from all classes before calculating precision and recall, thus giving more weight to larger classes and reflecting overall performance more accurately in imbalanced datasets.

Consequently, our comparisons focused solely on the results for the closed set data. This analysis enabled us to derive a macro F1 score, ensuring equal assessment across all methylation classes. This rigorous evaluation highlights SNUH-MC’s ability to navigate complex classification environments, demonstrating its ability to recognize unknown labels and perform effectively across a variety of methylation classes (Table 2).

**Table 2:** Performance comparison between SNUH-MC and DKFZ-MC

	Filtered test data set 1		Test data 1		Filtered test data set 2	
	F1-micro	F1-macro	F1-micro	F1-macro	F1-micro	F1-macro
SNUH-MC	0.932	0.919	0.611	0.528	0.9	0.531
DKFZ-MC	0.907	0.627	0.616	0.284	0.9	0.501

SNUH-MC, Seoul National University Hospital-Methylation Classifier; DKFZ-MC, Deutsches Krebsforschungszentrum-Methylation Classifier

We selected 767 probes for our analysis to ensure a fair comparison with the existing model, which specifically used 767 probes. This number was determined through optimization within the model’s training dataset, demonstrating that high performance could be achieved with fewer probes. Although we initially considered using 1000 probes, we opted for 767 to maintain consistency and isolate the impact of our feature selection process and the SMOTE algorithm. This decision allows us to accurately assess the effectiveness of our methodology while acknowledging that it may slightly reduce the performance of the SNUH-MC model. Future studies may investigate the effects of varying the number of probes on model performance.

To assess the impact of the SMOTE algorithm on the feature selection, we compared the performance of a model that used 767 probes selected through feature selection and was used for prediction with a Multi-Layer Perceptron (MLP) model 767-MC [13]. This comparison allowed us to evaluate how the feature selection process with 767 probes, followed by prediction using the MLP model, performed against a model where feature selection was optimized using the SMOTE algorithm. This detailed analysis provided valuable insights into how effectively the SMOTE algorithm could enhance feature selection and improve the overall classification model’s performance (Table 3). To compare with the 767-MLP models, which originally predicted only 91 classes, we modified these models to include an unknown class by assigning the lowest probability to the unknown class, effectively making them predict 92 classes.

**Table 3:** Performance comparison between SNUH-MC and MLP model

	Filtered test data set 1		Test data set 1	
	F1-micro	F1-macro	F1-micro	F1-macro
SNUH-MC (767 probes)	0.921	0.908	0.608	0.521
767-MC (767 probes)	0.901	0.892	0.587	0.387

SNUH-MC, Seoul National University Hospital-Methylation Classifier; 767-MC, A deep learning model utilizing 767 selected probes

### Noise data detection

To explore open-set recognition capabilities, 485 sarcoma samples with unknown labels were introduced, and a comparative analysis was performed. In this evaluation, we merged Test Data Set 2 with additional unknown label data, allowing us to observe the impact on the F1 score as the number of noise samples gradually increased (Supplementary Fig. 4).

The macro F1 score decreases, while the micro F1 score increases with additional noise samples, revealing important nuances in our classifier’s performance. Micro F1 score was more heavily influenced by performance in major classes, as it considered all samples equally. The increasing trend suggests that SNUH-MC maintains good performance on well-represented classes even when noise increases, indicating a certain degree of robustness for these classes.

Macro F1 score gives equal weight to all classes. Its decreasing trend indicates that performance in minority classes is more sensitive to noise, suggesting less robustness for these rarer tumor types.

This divergence in trends provides valuable insights. SNUH-MC demonstrates resilience in identifying and managing common tumor types, even in increasing noise. However, its performance on rarer tumor types is more susceptible to noise, highlighting an area for potential improvement.

### Comparison of SNUH-methylation classifier and DKFZ methylation classifiers through ‘decisions’

The classification models constructed by SNUH and DKFZ each provided two sets of results: DKFZ-MC generated the results for v11b4 and v12.5, while SNUH-MC yielded results using SoftMax and OpenMax. SoftMax generated results based on the 91 methylation classes of DKFZ-MC v11b4, whereas OpenMax produced 92 classes, including the ‘unknown’ cluster. The detailed information on SNUH cases is summarized in Supplementary Table 2.

**Fig. 2:**
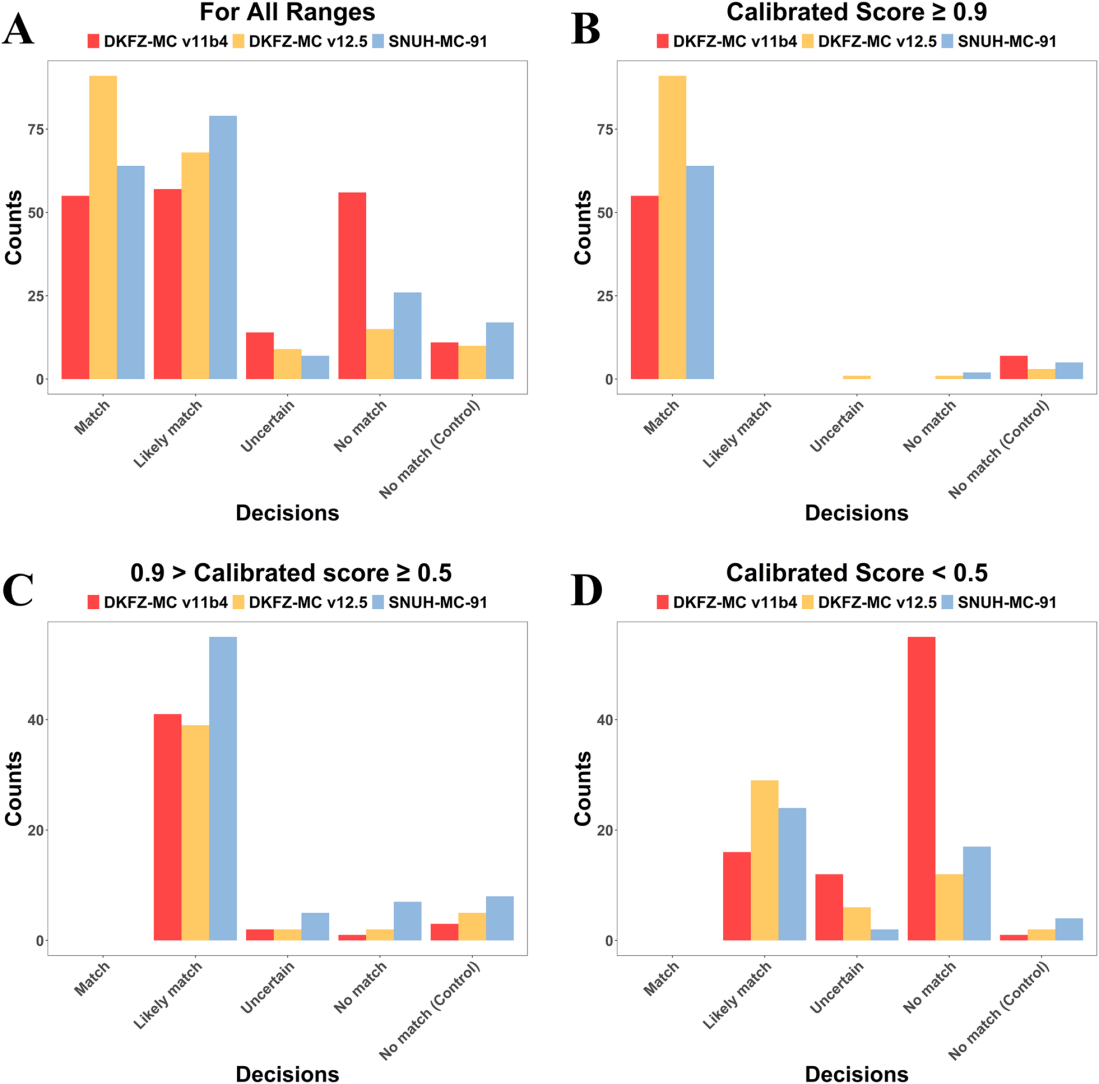
Comparison of SNUH-MC-91 and DKFZ-MC Decisions: This figure compares the decision results of SNUH-MC-91 and DKFZ-MC across different calibrated score ranges. The decisions are categorized as follows: **A** All ranges: Distribution of all decisions regardless of calibrated score **B** Score ≥ 0.9: High-confidence decisions, **C** 0.9 > Score ≥ 0.5: Medium-confidence decisions, **D** Score < 0.5: Low-confidence decisions “Match” indicates cases where the methylation classifier result (with score ≥ 0.9) is consistent with histopathology, clinical information, and NGS findings. This comparison illustrates the performance and agreement of both classifiers with other diagnostic methods across various confidence levels, providing insights into their reliability in neuropathological diagnosis. (SNUH-MC: Seoul National University Hospital Methylation Classifier; DKFZ-MC: Deutsches Krebsforschungszentrum Methylation Classifier; NGS: Next Generation Sequencing)

First, we compared and analyzed the results calculated from ‘SNUH-MC-SoftMax (referred to as SNUH-MC-91)’ and the results obtained from two versions of the DKFZ-MC. Initially, the evaluation involved quantifying the ‘Decisions’ made by each methylation classifier, as depicted in Fig. 2.

Across all score ranges, DKFZ-MC v12.5 exhibited the highest frequency of the ‘Match’ class for our cohort, followed by SNUH-MC-91 and DKFZ-MC v11b4. Regarding ‘Likely match’ probability, SNUH-MC-91 displayed the highest value, followed by DKFZ-MC v12.5 and v11b4. In cases classified as ‘No match,’ DKFZ-MC v11b4 had the highest incidence, followed by SNUH-MC-91 and DKFZ-MC v12.5. This comparative analysis provides insights into the distribution and frequency of the different classifications made by each methylation classifier across varying score ranges.

We compared the ‘Decisions’ results between DKFZ-MC v11b4 and v12.5 for 193 cases. Our multidisciplinary team, comprising neuropathologists, molecular biologists, and bioinformaticians, assessed each case using a consensus-based approach. This assessment involved comparing methylation-based classifications with histopathology, molecular markers, imaging features, and clinical presentations. Of the 193 cases: 98 (50.8%) were reclassified based on our integrated assessment criteria (Supplementary Fig. 5A). Among these, 47 (24.4%) were classified as ‘Match’, indicating high concordance between methylation results and other diagnostic methods. 38 (19.7%) were ‘Likely match’, 6 (3.1%) ‘Uncertain’, and 5 (2.6%) ‘No match’. 2 cases (1.0%) switched from v11b4 to v12.5, resulting in ‘No match (control)’. Notably, 56 cases initially classified as ‘No match’ in v11b4 were reclassified in v12.5:20 (10.4%) as ‘Match’, 20 (10.4%) as ‘Likely match’, 4 (2.1%) as ‘Uncertain’, and 2 (1.0%) as ‘No match (Control)’.

Of the 47 ‘Match’ cases, 17 (36.17%) were identified as novel subtypes by v12.5, including germ cell tumor_germinoma_KIT mutation (GCT_GERM_KIT), germ cell tumor_teratoma (GCT_TERA), neuroepithelial tumor_PATZ1 fusion-positive (NET_PATZ1), neuroepithelial tumor_PLAGL1 fusion-positive (NET_PLAGL1_FUS), and diffuse pediatric-type high-grade glioma_receptor tyrosine kinase 1A-type (pedHGG_RTK1A). This indicated the emergence of new subtypes recognized by the updated methylation classifier. These classification changes highlighted the evolving nature of classifications between DKFZ-MC v11b4 and v12.5, introducing novel subtypes and reassigning cases across different matching categories.

Comparing the SNUH-MC-91 classification model and DKFZ-MC v11b4, 69 out of 193 cases (35.8%) were reclassified with SNUH-MC-91. Due to these changes, 17 cases changed to ‘Match’ (8.8%), 34 cases to ‘Likely match’ (17.6%), 4 cases to ‘Uncertain’ (2.1%), 5 cases to ‘No match’ (2.6%) and 9 ‘No match’ cases were control (4.7%).

**Fig. 3:**
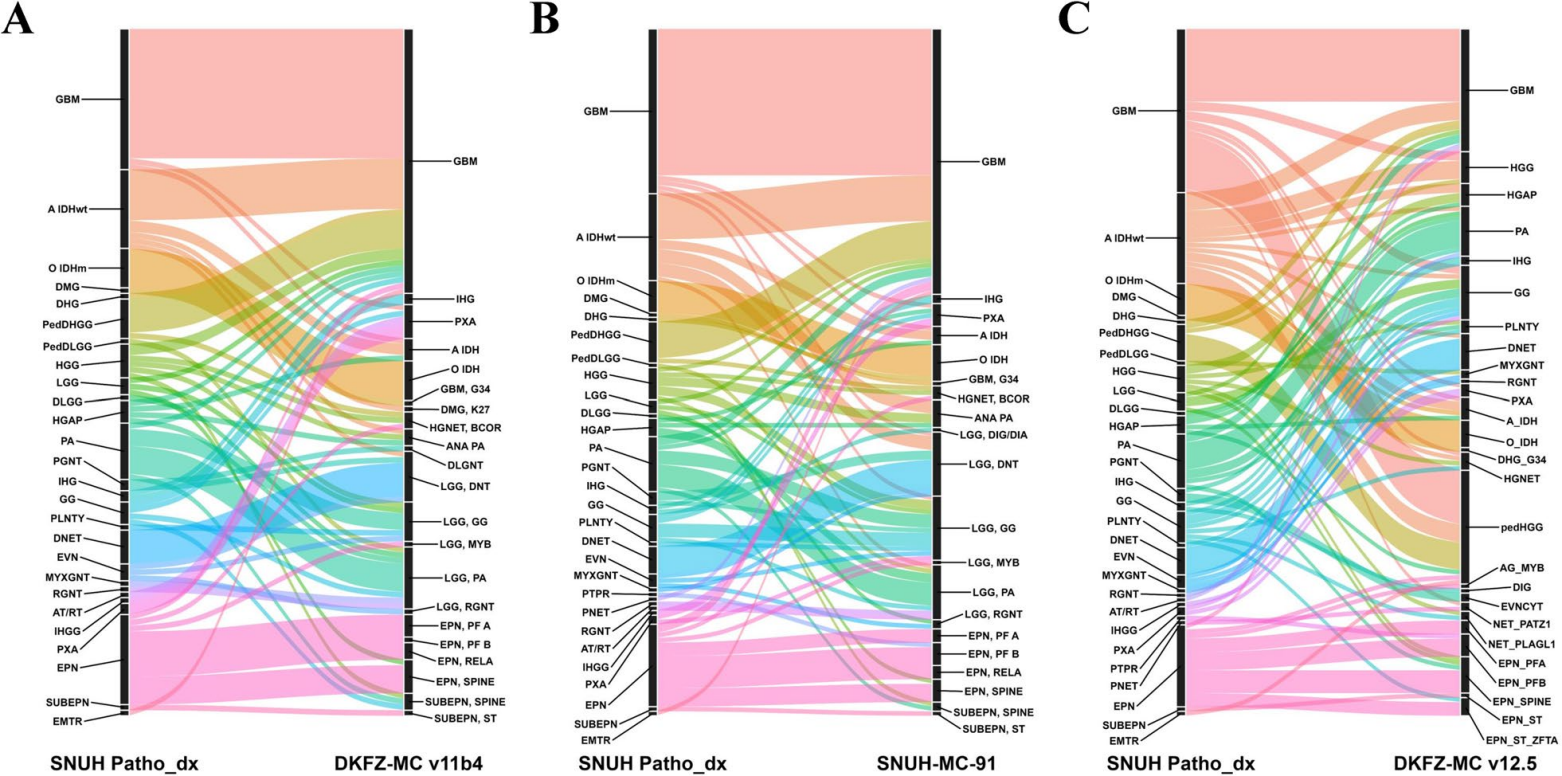
Comparison of methylation-based classification results for gliomas, glioneuronal, and neuronal tumors against initial SNUH pathological diagnoses. **A** DKFZ-MC v11b4 results, **B** SNUH-MC-91 results, and **C** DKFZ-MC v12.5 results. Each plot displays methylation-based classifications (right) compared to initial SNUH pathological diagnoses (left). Colors are consistent across plots and correspond to SNUH pathological diagnoses. DKFZ-MC v11b4 and SNUH-MC-91 use the same 91 methylation class labels, while DKFZ-MC v12.5 introduces new labels from its expanded 170 methylation classes. Novel tumor types in v12.5 (e.g., pedHGG, NET_PATZ1) reflect recent advances in molecular tumor classification. Detailed results are available in Supplementary Table 2 (SNUH-MC: Seoul National University Hospital-Methylation Classifier; DKFZ-MC: Deutsches Krebsforschungszentrum-Methylation Classifier)

**Fig. 4:**
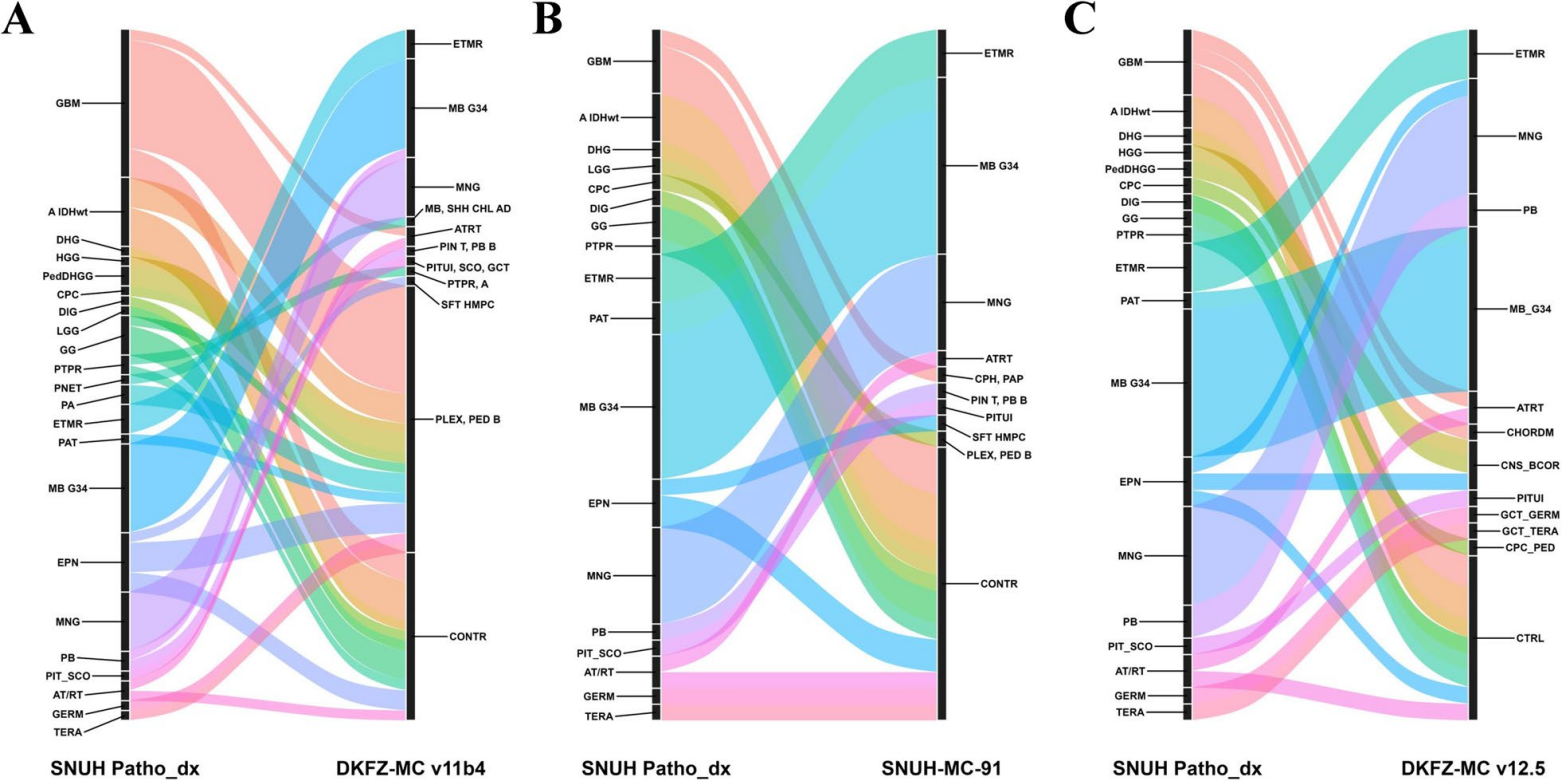
Comparison of methylation-based classification results for embryonal, pineal, meningiomas, and other CNS tumors against SNUH pathological diagnoses. **A** DKFZ-MC v11b4 results, **B** SNUH-MC-91 results, and **C** DKFZ-MC v12.5 results. Each plot displays methylation-based classifications (right) compared to initial SNUH pathological diagnoses (left). DKFZ-MC v11b4 and SNUH-MC-91 use the same 91 methylation class labels, while DKFZ-MC v12.5 introduces new labels from its expanded 170 methylation classes. Novel tumor types in v12.5 reflect recent advances in molecular tumor classification as per the 2021 WHO Classification of Tumors of the Central Nervous System. Detailed results are available in Supplementary Table 2 (SNUH-MC: Seoul National University Hospital-Methylation Classifier; DKFZ-MC: Deutsches Krebsforschungszentrum-Methylation Classifier)

When comparing SNUH-MC-91 to DKFZ-MC v12.5, 89 out of the 193 cases (46.1%) exhibited new assignments in DKFZ-MC v12.5. Among these, 45 cases were reclassified into ‘Match’ (23.3%), 30 cases into ‘Likely match’ (15.5%), 6 cases into ‘Uncertain’ (3.1%), and 7 into ‘No match’ (3.4%), and 1 case into ‘No match (control)’ (0.5%). Supplementary Fig. 5B and Supplementary Table 3–5 provide detailed information regarding each reclassification. Additionally, Figs. 3 and 4 depict the changes in diagnoses comparing the initial pathological diagnosis from SNUH with each classifier.

This comparison highlighted the differences in reclassifications between SNUH-MC-91 and the DKFZ-MCs versions (v11b4 and v12.5) under different classification models for the same set of cases.

Unlike SNUH-MC-91, SNUH-MC-OpenMax (hereafter SNUH-MC-92) results include the ‘unknown’ cluster. When DKFZ-MC v11b4 was used as a reference, a significant number of cases (105 cases, 54.4%) were categorized as ‘unknown.’

The categorization into ‘unknown’ was due to six main causes:

- Low scores in both v11b4 and v12.5 (31/105, 29.5%)
- Low scores in v11b4 & Novel class in v12.5 (v12.5 scores > 0.87, 27/105, 25.7%)
- Low scores in both v11b4 and v12.5 & Novel class in v12.5 (v12.5 scores < 0.72, 13/105, 12.4%)
- Low score in v11b4 (11/105, 10.5%)
- Novel subtype in v12.5 (8/105, 7.6%)
- Inexplicable (15/105, 14.3%)

In the classification results derived from SNUH-MC-92, most of the ‘unknown’ cases found clarity based on their calibrated scores. However, explaining the 15 ‘inexplicable’ cases was challenging. To gain insight, these 15 cases were visualized using t-distributed stochastic neighbor embedding (t-SNE) plots (Supplementary Fig. 6, Supplementary Table 6). Upon closer inspection, their locations were noticeably outside the cluster boundaries. SNUH-MC-92 classified these cases as ‘unknown’ because they were outside the central cluster of training samples.

Further analysis revealed that 88 out of 193 cases (45.6%) were classified based on the existing 91 classes (Supplementary Table 7). Within this subset, 72 cases (81.8%) were classified consistently or similarly across v11b4, v12.5, SNUH-MC-91, and SNUH-MC-92 classifiers (shown in blue). 12 cases (13.6%) were cases where v11b4 could not classify correctly, but the results from v12.5 and SNUH-MC were in good agreement (shown in orange). 4 cases (4.6%) were cases where only the v12.5 classifier gave reasonable results (shown in pink); The integrated histopathological diagnoses of these cases were ‘GBM, IDH-wt or HGAP’, ‘pilocytic astrocytoma with *BRAF* fusion’, ‘diffuse low-grade glioma with *LHFPL3::BRAF* fusion’, and ‘no available information’.

These findings demonstrated that SNUH-MC-91 is consistent with DKFZ-MC v12.5 despite its configuration based on DKFZ-MC v11b4 reference data. Moreover, the ‘unknown’ categorization in SNUH-MC-92 originates from various reasons, shedding light on the complexities and reasons behind specific certain classifications. The subset analysis detailed the consistency and discrepancy among classifications across different methylation classifiers and versions.

### Can methylation profiling aid pathological diagnosis or not?

Methylation profiling can serve as a powerful tool, especially in scenarios where tumors exhibit similar morphology but differ at a molecular level. Among the cases challenging to diagnose using conventional pathology methods and the DKFZ-MC v11b4, 23 cases yielded similar or identical results between SNUH-MC-91 and DKFZ-MC v12.5, which were deemed reasonable (Supplementary Table 8). This exemplifies SNUH-MC’s strong performance, considering its basis on DKFZ-MC v11b4 reference data.

**Fig. 5:**
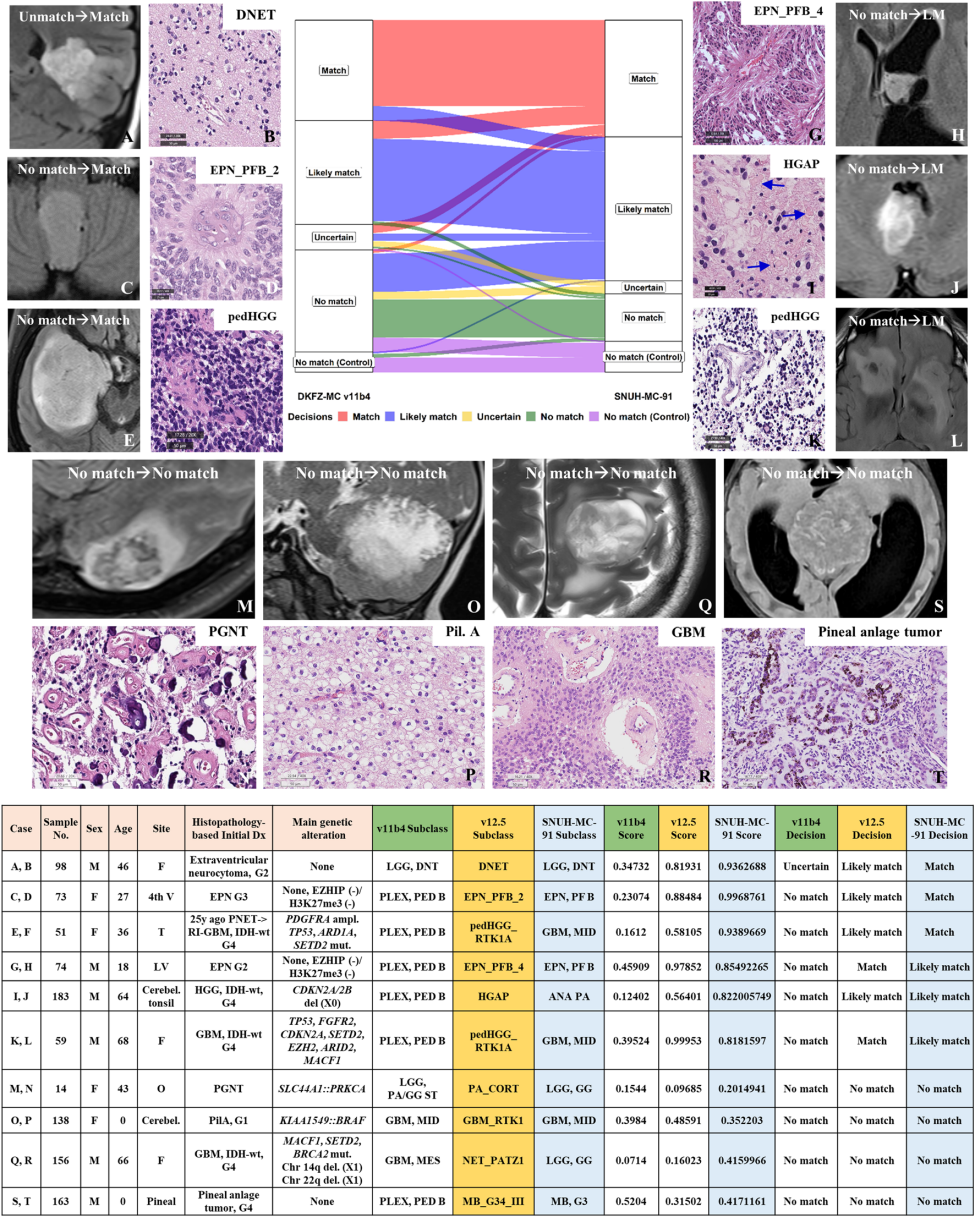
Changes in ‘Decisions’ from DKFZ-MC v11b4 to SNUH-MC-91. This figure illustrates the reclassification of 69 cases from DKFZ-MC v11b4 to SNUH-MC-91. Each case is labeled as “Uncertain → Match,” “No match → Match,” “No match → Likely match (LM),” or “No match → No match.” Each case is presented with MRI (T2-weighted FLAIR) and histopathological (H&E) images. Final diagnoses are provided as subheadings for each image set. Tables show detailed information including main genetic alterations obtained from NGS studies with brain tumor-targeted gene panel (SNUH FiRST brain tumor panel). The final diagnosis of each case is DNET **A**, **B**, EPN_PFB **C**, **D** & **G**, **H**), PedHGG **E**, **F** & **K**, **L**, and HGAP **I**, **J**. In Figure **I**, Rosenthal fibers (blue arrows) are evident. The last four cases were not matched by all methylation classifiers, including our version, which are PGNT with *SLC44A1::PRKCA* fusion **M**, **N**, pilocytic astrocytoma with *KIAA1549::BRAF* fusion **O**, **P**, GBM, IDH-wt **Q**, **R** and pineal anlage tumor **S**, **T**. Cases that remained unchanged in the ‘Decisions’ were also included in this plot (DKFZ-MC: Deutsches Krebsforschungszentrum-Methylation Classifier; SNUH-MC: Seoul National University Hospital-Methylation Classifier, DNT: low-grade glioma, dysembryoplastic neuroepithelial tumor, EPN: Ependymoma, PNET: primitive neuroectodermal tumor, HGG: high-grade glioma, G3, G4: grade 3, grade 4, IDH-wt, PLEX, PED B: choroid plexus tumor, pediatric type B, pedHGG_RTK1A: Diffuse pediatric-type high-grade glioma, receptor tyrosine kinase type 1A, HGAP: high-grade astrocytoma with piloid feature, ANA PA: anaplastic pilocytic astrocytoma, LGG, PGNT: papillary glioneuronal tumor, Pil. A: pilocytic astrocytoma, ampl: amplification; mut.: mutation, Underbar size of the HE figures: B: 25 μm, D, F, K, N-T: 50 μm, G: 100 μm, I: 20 μm)

Figure 5 illustrated some cases, including histopathological images: The ‘Decisions’ changed from ‘No match’ to ‘Match’ (category 1), ‘Uncertain’ to ‘Likely match’ (category 2), and ‘No match’ to ‘Likely match’ (category 3). However, there were also four cases where all classifier versions, including the most recent versions, could not properly differentiate the tumor subtypes (‘No match’ to ‘No match’, category 4). Our firsthand experiences illustrated scenarios where methylation classifiers, particularly SNUH-MC, play a crucial role in improving diagnostic accuracy (categories 1–3), as well as cases where their current limitations are evident (category 4).

**Fig. 6:**
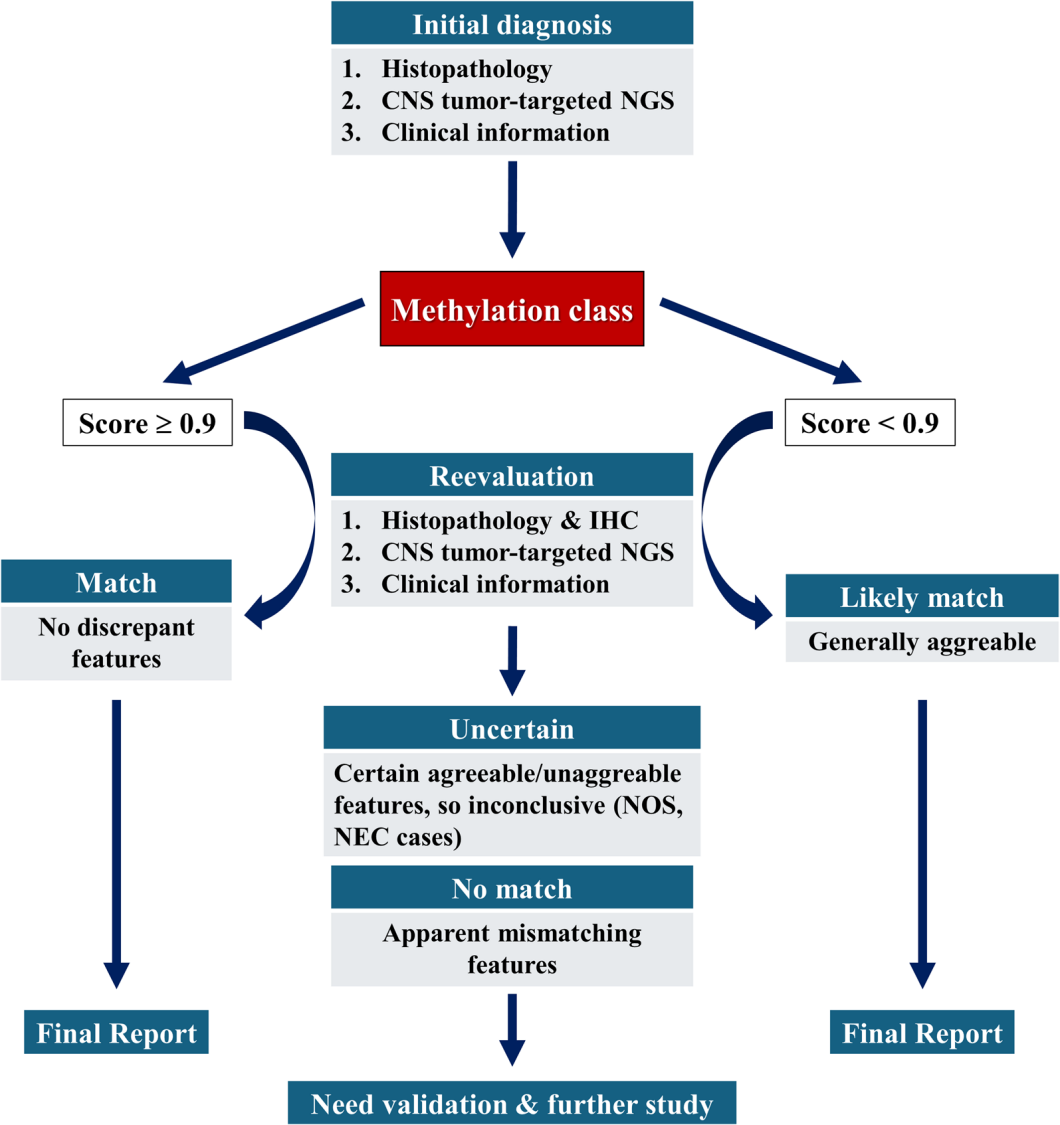
Integrated diagnostic approach for CNS tumors. This figure illustrates a comprehensive diagnostic process for CNS tumors, combining traditional histopathology with advanced molecular techniques. The diagnostic workflow begins with the initial diagnosis based on histopathology, NGS results, and clinical information. If this aligns with the methylation class (MC score > 0.9), or if there is general agreement despite an MC score < 0.9, final report is issued. In cases of discordance, (classified as Uncertain or No match), further validation is required, including reevaluation of histopathology, immunohistochemistry (IHC), NGS results, and clinical data. Uncertain cases may include tumors previously labeled as NOS (Not Otherwise Specified) or NEC (Not Elsewhere Classified). This approach enhances diagnostic accuracy

In summary, methylation profiling can significantly enhance pathological diagnosis, particularly in cases with ambiguous morphology or molecular heterogeneity. However, there are instances where methylation classifiers may not provide a definitive diagnosis, highlighting the importance of integrating multiple diagnostic modalities for accurate tumor classification (Fig. 6).

## Discussion

We developed a methylation classification tool named SNUH-MC, employing the SMOTE algorithm to address data imbalance and integrating OpenMax with an MLP, making it distinct from previous algorithms. Our findings show SNUH-MC’s strong ability to accurately classify unknown data compared to existing methods. SNUH-MC utilized the SMOTE algorithm to address data imbalance and integrated OpenMax within a Multi-Layer Perceptron to prevent labeling errors in low-confidence diagnoses. This strengthens its reliability as a disease classifier, prioritizing both data balance and precise diagnosis. Additionally, we introduced calibrated score ranges and established criteria called ‘Decisions,’ providing a strategic framework for researchers to assess methylation profiling outcomes.

Among the 23 samples that the DKFZ-MC v11b4 did not classify correctly, cases initially labeled as ‘choroid plexus tumor, subclass pediatric B (PLEX, PED B)’ were later classified in v12.5 as ‘Diffuse pediatric-type high-grade glioma (pedHGG).’ However, SNUH-MC-91 results labeled these cases as ‘GBM, IDH-wt.’ SNUH-MC-91 refers to labels from v11b4 and does not include representation for the ‘pedHGG’ cluster, thus supporting the validity of the ‘GBM’ classification. Moreover, in our cases, 14 out of 193 (7.3%) cases initially identified as GBM in v11b4 were later reclassified as pedHGG in v12.5. Because pedHGG and GBM share molecular and morphological features this result was thought reasonable. Moreover, this change in methylation class was aligned with the other literatures [23]. Neither DKFZ-MC v11b4 nor SNUH-MC-91 includes the pedHGG classification. Both of these classifiers are based on the same dataset and do not incorporate the newer classifications found in later versions. SNUH-MC-91 was developed based on DKFZ v11b4, so it has the same set of tumor classifications. As we mentioned in method section, we could not have access to the dataset used for DKFZ v12.5 or v12.8, which limits our ability to include these newer classifications in our SNUH-MC-91. Similarly, cases initially classified as ‘choroid plexus papilloma_pediatric type B (PLEX, PED B)’ in v11b4 were later recategorized as ‘ependymoma_posterior fossa group B (EPN_PFB)’ in v12.5. These cases were consistently classified as ‘EPN, PFB,’ in the SNUH-MC-91 classification. In summary, the observed enhancement in reclassification for our methylation classifier mirrors the performance of DKFZ-MC v12.5, benefiting from an extensive database with over 90 k CNS tumor reference data compared to the previous version, DKFZ-MC v11b4. However, to substantiate these reclassifications, we propose the following approach: Conduct comprehensive histopathological re-evaluation and supplementary molecular analyses to corroborate tumor identities. Incorporate longitudinal clinical data to assess tumor behavior in concordance with the revised classification. Perform a systematic review of contemporary literature to identify analogous reclassification patterns and their clinical implications. While the expanded dataset in v12.5 may potentially enhance diagnostic precision, it does not inherently guarantee improved accuracy across all cases.

We explored the classification results of SNUH-MC-92, achieving clarity for most ‘unknown’ cases through calibrated scores. However, 15 cases labeled as ‘inexplicable’ underwent visual analysis using t-SNE plots, revealing subtle deviation from the central cluster of training samples despite being classified as ‘unknown’ by SNUH-MC-92 (Supplementary Fig. 5).

Our work with SNUH data highlights its advantages for researchers, particularly in uncovering diverse molecular features through methylation profiling. For example, distinct clusters have emerged in tumors with fusion genes like *PATZ1, BCOR/BCORL1, ZFTA*, and *PLAGL1* [4, 24–26]. Methylation data have also provided insight into critical elements such as CNVs and SNPs, which play a crucial role in tumor classification. Classes characterized by specific genetic alterations, which are often associated with distinct methylation patterns, include *MYCN* amplification & isochromosome 17q in medulloblastoma groups 3 and 4, loss of chromosome 22 in atypical teratoid/rhabdoid tumor, *MYB/MYBL1* alterations in angiocentric glioma, and *C19MC::TTYH1* fusion in embryonal tumor with multilayered rosettes [27–31].

These genetic changes, detectable through various molecular methods, are frequently correlated with specific methylation signatures, allowing methylation profiling to serve as a surrogate marker for these molecular features.

Although we did not specifically measure or analyze MGMT promoter methylation status from our methylation array data, the methylation data also allowed us to predict the *MGMT* promoter methylation status [32]. These unique properties highlight how epigenetics has emerged as a powerful tool for CNS tumor researchers, providing rich insights into tumor biology.

While methylation profiling is a powerful tool for tumor classification, there are limitations to its application. Accurate methylation profiling requires high-quality and quantity DNA samples, typically at least 0.5ug. Obtaining precise methylation data becomes challenging when dealing with small tumor tissue sizes, especially in biopsy samples, or subpar DNA quality. It is crucial to check the quality and integrity of DNA samples before starting the experiment. FFPE DNA samples often undergo degradation due to chemical fixation and treatment. Over-fixation is a recognized factor contributing to reduced quality and quantity during DNA extraction [33]. Storage conditions, including temperature, humidity, and duration, significantly impact DNA integrity and quantity [34, 35]. Therefore, researchers should thoroughly consider these factors before performing experiments and may need to conduct additional FFPE DNA restoration assays to address subpar DNA quality issues [36].

After generating methylation data, researchers closely examine several factors: 1) ensuring gender consistency with the patient, 2) ensuring that CNV plots derived from methylation data are noise-free, and 3) verifying that expected results match the data [22]. For example, if neuropathologists expected MC of GBM with FGFR3-TACC3 fusion, but the MC often matched with ganglioglioma with v12.5 [37], careful validation and investigation are essential. While valuable, methylation classifiers have limitations: They cannot classify all types of brain tumors. They may produce erroneous matches even with high scores.

Methylation profiling poses a significant challenge due to the potential for uncertain or indeterminate results, especially in molecularly diverse CNS tumors like gliomas. Inadequate representation of rare tumor types in classifier training datasets may lead to inconclusive or conflicting results. Therefore, interpreting methylation data requires integration with clinical and pathological findings for accurate diagnosis. Continuous updating of training datasets is crucial to improve classifier performance across diverse tumor types,

In methylation studies, the emergence of unknown clusters not matching existing methylation classes requires thorough investigation. For ‘No match’ cases, a comprehensive approach should include repeating methylation profiling, conducting thorough histopathological reviews with additional stains, performing supplementary molecular tests, reviewing clinical data and imaging features, consulting experts for challenging cases, and considering the possibility of novel tumor entities.

For all tumor samples, reevaluation is essential if the calibrated score is less than 0.9 or noticeably low, even when using regions with high tumor cell content (typically > 70%). This is because tumor cell purity can vary within samples, particularly in infiltrative tumors like diffuse gliomas. In such cases, reassessing DNA quality, re-examining histology to confirm tumor content, and considering repeat methylation profiling may be necessary to ensure accurate classification results.

A ‘No match’ result may stem from tissue heterogeneity or low tumor purity, particularly in low-grade tumors. Addressing this requires a comprehensive process involving expert pathological evaluation, clinical observations, bioinformatic data, and literature reviews. A literature review can help resolve “no match” results by identifying recently described tumor entities, finding reports of similar ambiguous cases, and discovering new molecular markers relevant to the case. This process can provide context for interpreting challenging results and suggest alternative approaches for classification. However, conducting thorough literature reviews adds to the workload of the experts involved, requiring a balance between comprehensive analysis and practical considerations.

Methylation profiling precision depends on methodology and reference databases, impacting accuracy [12, 13]. Limited access to methylation tests in certain healthcare settings due to cost and resource constraints is an issue. Profiling CNS tumors effectively requires comprehensive reference databases, posing challenges for unique methylation patterns. The challenge with reference databases for CNS tumor methylation profiling is primarily related to incomplete coverage, not conflicting data. Continuous updating of reference databases is crucial to capture the full spectrum of CNS tumor heterogeneity and improve classification accuracy, especially for uncommon or newly discovered tumor entities.

Limitations in methylation data can complicate subtype differentiation, necessitating advanced analytics for accurate distinctions. The main limitations in CNS tumor methylation profiling include data imbalance: Underrepresentation of rare tumor types in reference databases, lack of reference data for newly discovered or reclassified tumor entities, potential misclassification of similar tumor subtypes, and evolving tumor classifications that may outpace database updates.

Staying updated on profiling advances is crucial, as it may improve the identification of previously unmatchable samples. Acknowledging and addressing these limitations is essential when using methylation data for pathological diagnoses.

Ongoing research in AI and machine learning is expanding into various aspects of neuropathology, including methylation-based diagnostics. While our focus is on DNA methylation profiling, it’s worth noting that complementary AI approaches are being developed for whole slide image analysis and gene mutation prediction [38–40]. Additionally, machine learning models could help identify correlations between methylation patterns and visual features in pathology slides, potentially uncovering new biomarkers or diagnostic criteria. Challenges in developing these integrated approaches include data heterogeneity across institutions and the need for large, well-annotated datasets. However, as both methylation profiling and AI-based image analysis continue to advance, their combination may lead to more accurate and efficient CNS tumor diagnostics, potentially reducing pathologists’ workload and improving diagnostic precision [41].

While possible, the focus should be on identifying the most variable sites between tumor subtypes rather than simply increasing the number of sites analyzed. This can be achieved through methods like methylation sequencing, single-cell analysis, and GWAS studies. Combining methylation data with traditional pathology, SNP, CNV, and fusion data could create more comprehensive and robust classification models. Such multi-modal approaches could leverage the strengths of each data type, potentially improving accuracy in challenging cases.

The integration of methylation profiling with traditional histopathology and NGS in pathology institutions significantly enhances the final pathological diagnosis, influencing clinical treatment decisions [22, 42, 43].

## Conclusions

This study represents a novel methylation classification tool called SNUH-MC that addressed several key challenges in accurate brain tumor classifications. By employing advanced techniques like the SMOTE algorithm for data imbalance and OpenMax integration for open-set recognition, SNUH-MC demonstrates improved performance compared to existing methods, particularly in handling unknown or noisy data samples. To demonstrate the robustness and improvement of our classification method, we should correlate MC with patient outcomes (survival rates, progression-free intervals, treatment responses), assess the impact of reclassifications on patient management and outcomes, perform external validation using independent datasets, conduct longitudinal studies to evaluate long-term predictive accuracy and integrate methylation-based classification with established molecular markers. However, we could not perform these analyses due to limitations in our study design and available data. This represents a limitation of our current work and highlights important directions for future research to fully validate the clinical utility of our approach.

Furthermore, the introduction of calibrated score ranges and the ‘Decisions’ criteria offer a strategic framework for researchers to assess methylation profiling outcomes, facilitating a deeper understanding of the classifier’s performance and the underlying reasons behind specific classifications.

This paper contributes to an advanced methylation classification algorithm, providing comprehensive insights based on data experience, spanning sample preparation to the interpretation of results obtained from the methylation classifiers.

## Supplementary Information

Additional file 1. 

Additional file 2. 

## Data Availability

The SNUH-MC Software is publicly available on GitHub under the MIT license and can be found at https://github.com/jaeminjj/SH_MC. The data reported in this paper have been deposited in KArray, part of the Korea BioData Station, managed by the Korea Bioinformation Center (KAP240833, https://kbds.re.kr/KArray).
