# Supplementary material for: SNUH methylation classifier for CNS tumors
Source: Clin Epigenetics. 2025 Mar 12;17:47. doi: 10.1186/s13148-025-01824-0 (PMC11905536; doi:10.1186/s13148-025-01824-0)
Supplement: Supplementary file 1 — Additional file 1. [file 13148_2025_1824_MOESM1_ESM.docx]

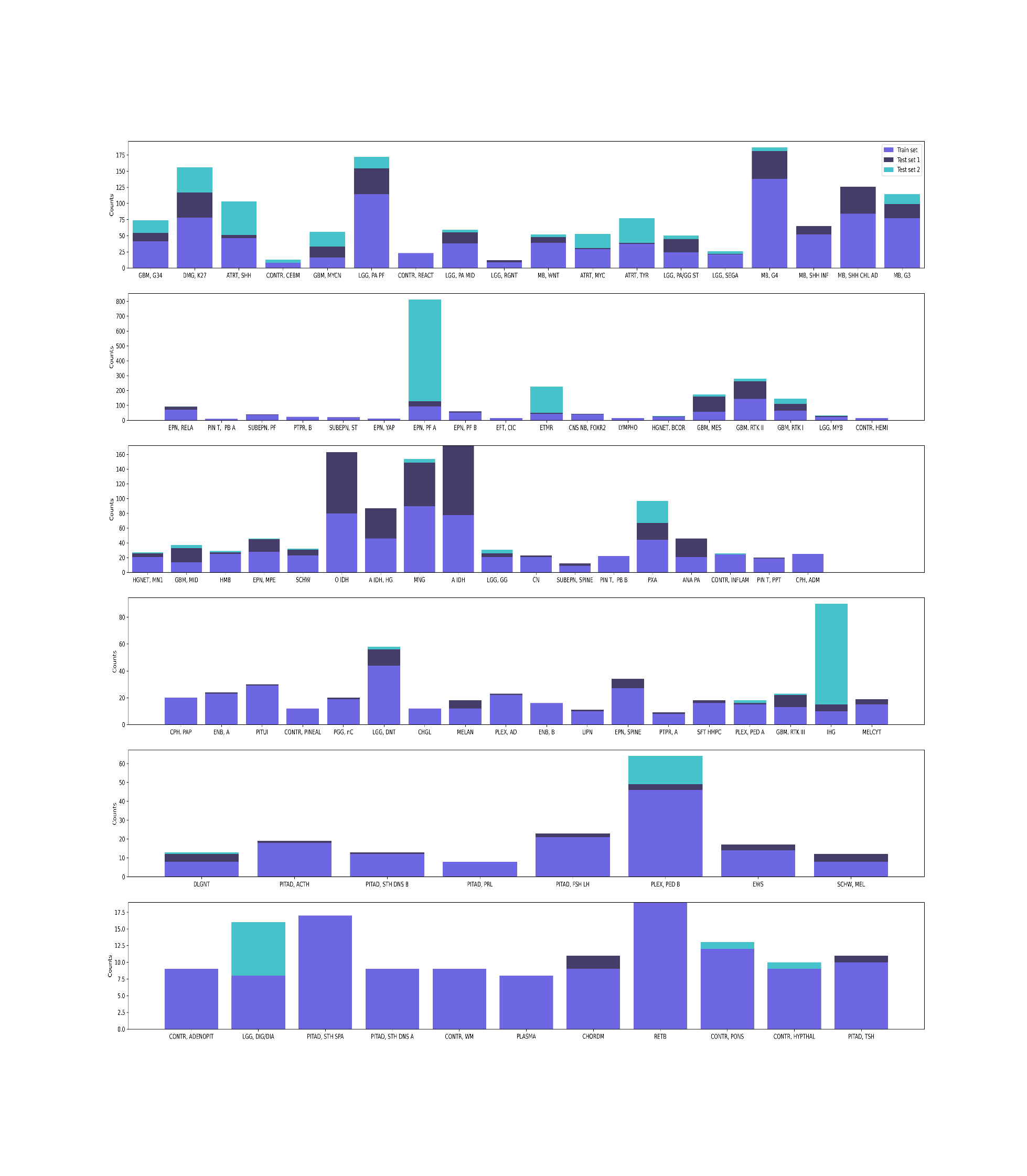


**Supplementary Figure 1. Bar plots depicting the distribution of 91 methylation classes among the samples.**


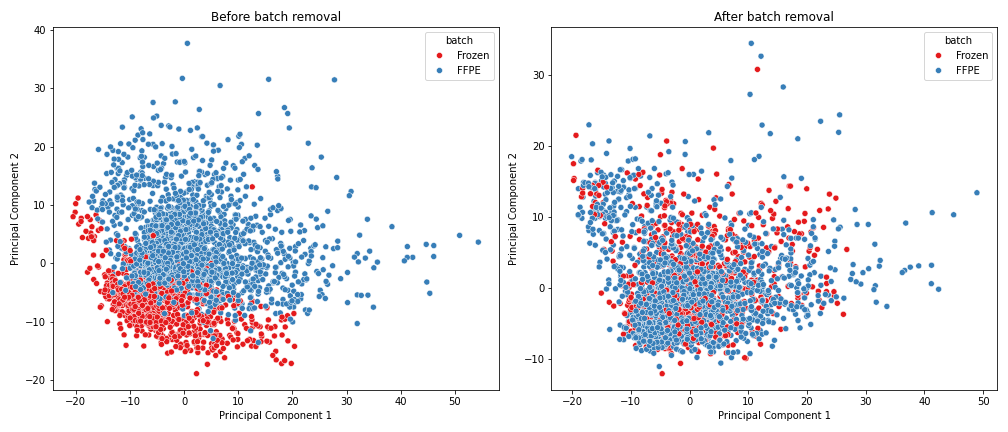


**Supplementary Figure 2. Comparison of PCA results before and after Batch Effect Removal** (PCA: Principal Component Analysis)


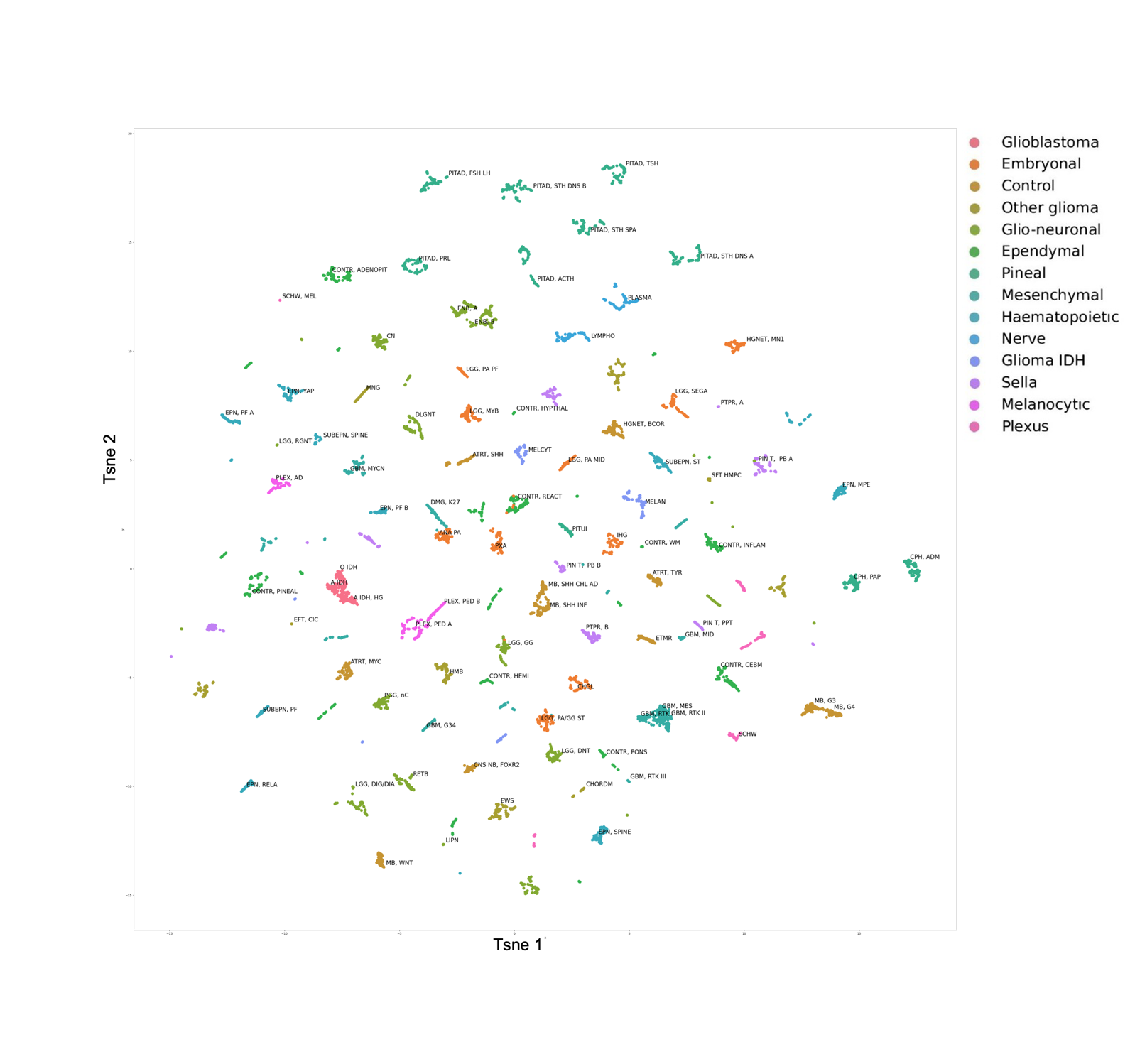


**Supplementary Figure 3. t-SNE plot of oversampling.** (t-SNE: t-distributed stochastic neighbor embedding)
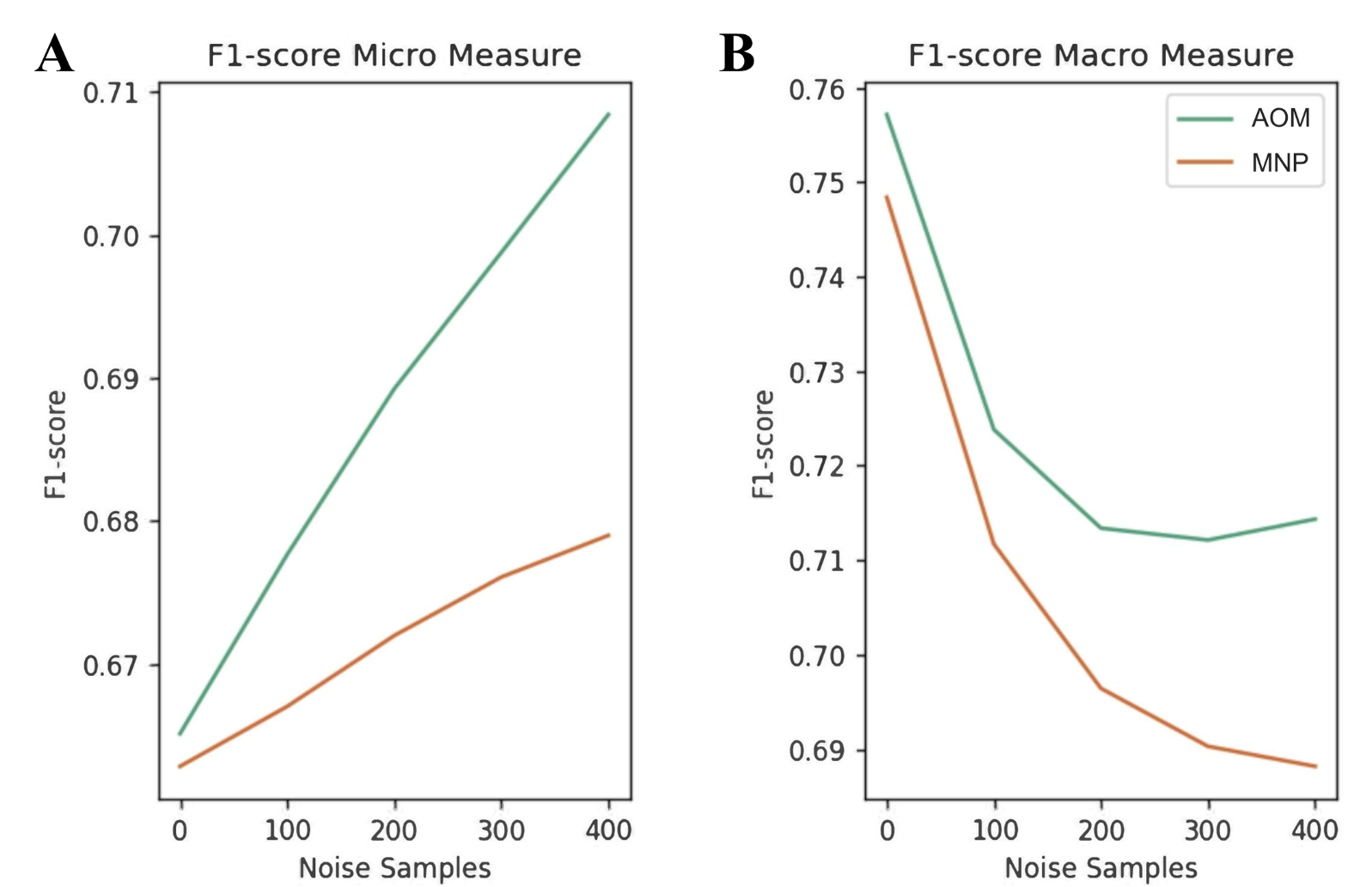


**Supplementary Figure 4. Comparison of classifier accuracy in the presence of noise samples.**


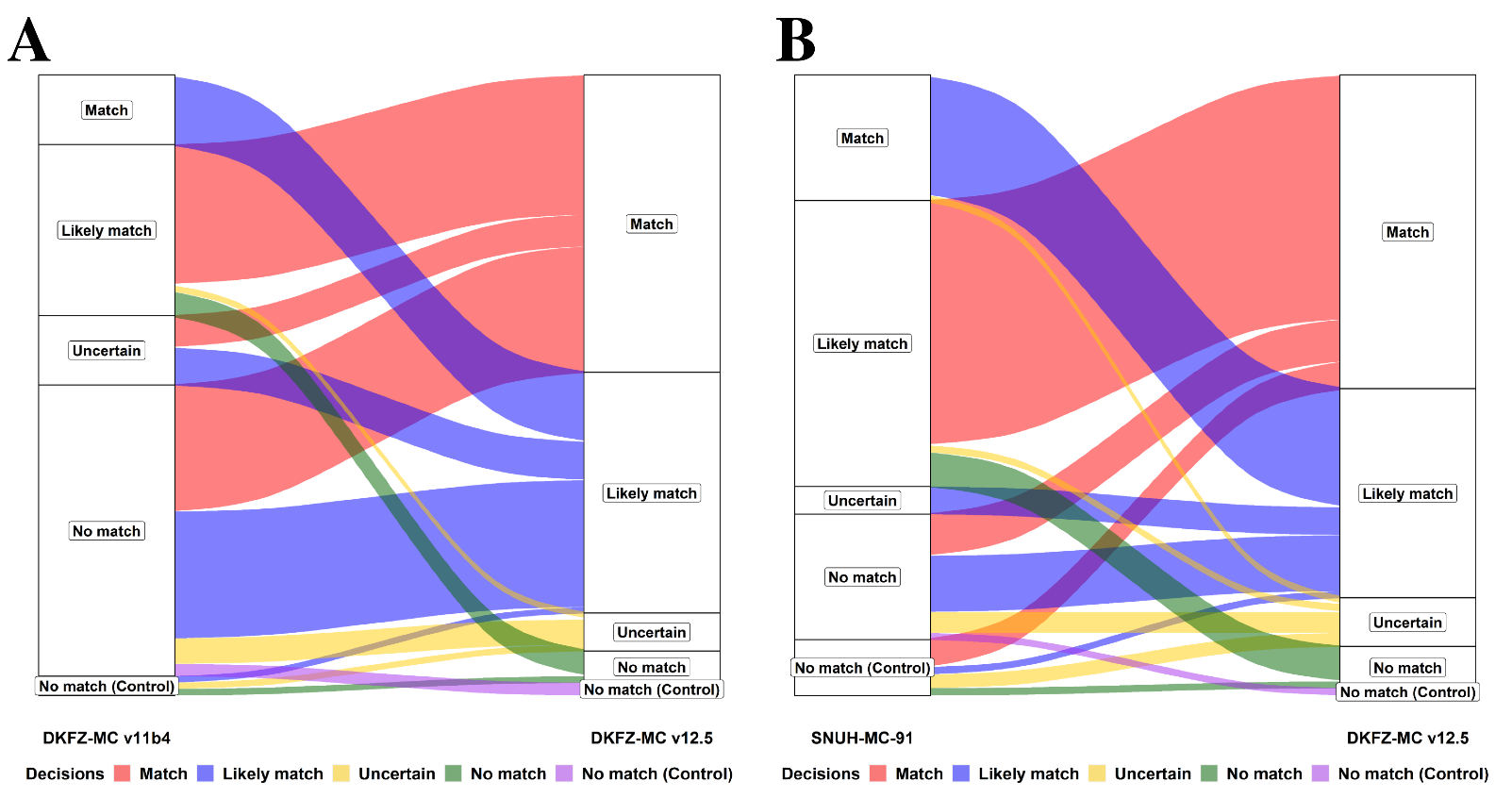


**Supplementary Figure 5. Plots illustrating changes in 'Decision' through the comparison of each of the three methylation classifiers.** A) DKFZ-MC v11b4 vs v12.5 and B) SNUH-MC-91 vs DKFZ-MC v12.5. Detailed information was provided in Supplementary Table 5 including the alluvial plot of Figure 5. (SNUH-MC: Seoul National University Hospital - Methylation Classifier; DKFZ-MC: Deutsches Krebsforschungszentrum - Methylation Classifier)


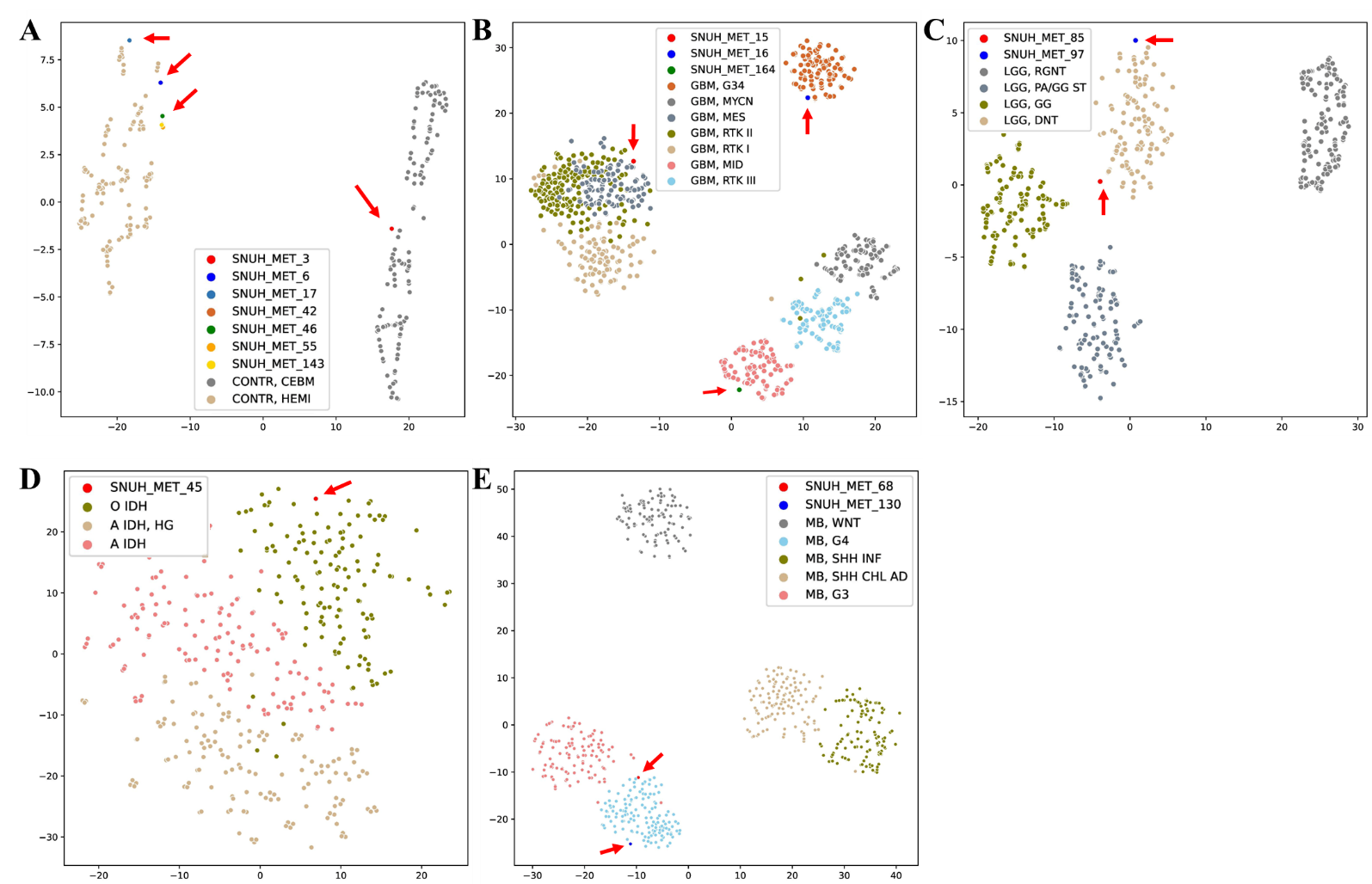


**Supplementary Figure 6. t-SNE plot of 15 ‘Inexplicable’ cases classified as ‘unknown’ in SNUH-MC-92.** t-SNE plot of 15 'inexplicable' cases classified as 'unknown' in SNUH-MC-92. Each arrow in the plot indicates one of these cases, which were labeled as 'unknown' due to their inability to fit into any predefined classification, even after analysis. The plots show samples corresponding to the existing v11b4 labels: A) CONTR, B) GBM, C) LGG, D) O IDH, and E) MB. Detailed information on these cases is provided in Supplementary Table 6. (CONTR: Control; GBM: Glioblastoma; LGG: Low grade glioma; O IDH: Oligodendroglioma, *IDH-*mutant; MB: Medulloblastoma)
